# Identification and Semi-Synthesis of 3-*O*-Protocatechuoylceanothic Acid, a Novel and Natural GPR120 Agonist †

**DOI:** 10.3390/molecules24193487

**Published:** 2019-09-26

**Authors:** Changjin Lim, Jung Gyu Park, Kyo Bin Kang, Young-Ger Suh

**Affiliations:** 1 College of Pharmacy, CHA University, 120 Haeryong-ro, Pocheon 11160, Gyeonggi-do, Korea; koryoi@cha.ac.kr; 2 College of Pharmacy and Research Institute of Pharmaceutical Sciences, Seoul National University, 1 Gwanak-ro, Gwanak-gu, Seoul 08826, Korea; ddhelix@lgchem.com; 3 Research Institute of Pharmaceutical Sciences, College of Pharmacy, Sookmyung Women’s University, Seoul 04310, Korea; kbkang@sookmyung.ac.kr

**Keywords:** G protein-coupled receptor, GPR120, agonist, 3-*O*-protocatechuoylceanothic acid, semi-synthesis

## Abstract

The identification and three step synthesis of 3-*O*-protocatechuoylceanothic acid, a novel and natural GPR120 agonist, is described. This ceanothane-type triterpenoid was identified from the components of *Ziziphus jujuba* roots and was found to be a new GPR120 agonist with a novel structure. We synthetically converted ceanothic acid, which does not have GPR120 agonist activity, into 3-*O*-protocatechuoylceanothic acid in three steps. In addition, we present the corrected NMR spectrum of 3-*O*-protocatechuoylceanothic acid based on our synthesis.

## 1. Introduction

**Figure 1:**
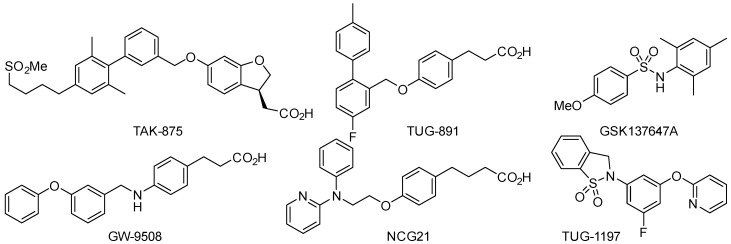
Structures of the reported GPR120 agonists.

In recent years, G protein-coupled receptors (GPCRs), which are activated by fatty acids and their metabolites, have evolved as novel molecular targets for the treatment of metabolic diseases [1]. GPR120, first discovered in 2005, is one of the GPCRs that is activated by an omega-3 fatty acid [2,3,4,5]. The activation of GPR120 increases glucose transportation from adipocytes to somatic cells resulting in a decrease of the glucose levels in obese/diabetic rats and also inhibits inflammatory responses of macrophages, thus imparting an anti-inflammatory effect [4,5,6]. Because a number of metabolic diseases, including type-2 diabetes and insulin resistance, are closely related to the chronic inflammatory response, these pharmacological functions of GPR120 have recently encouraged many pharmaceutical companies to choose GRP120 as a novel target for the treatment of metabolic diseases [2,7]. Although the activation of GPR120 is considered important for the treatment of metabolic diseases, only a few GPR120 agonists [5,8,9,10,11,12,13,14,15,16] have been reported so far (Figure 1). In our pursuit of developing a novel GPR120 agonist, we identified a natural GPR120 agonist with a novel skeletal backbone. The identified natural agonist, 3-*O*-protocatechuoylceanothic acid (**1**), could be synthesized in three steps from a triterpene that had no GPR120 agonist activity.

## 2. Results and Discussion

**Figure 2:**
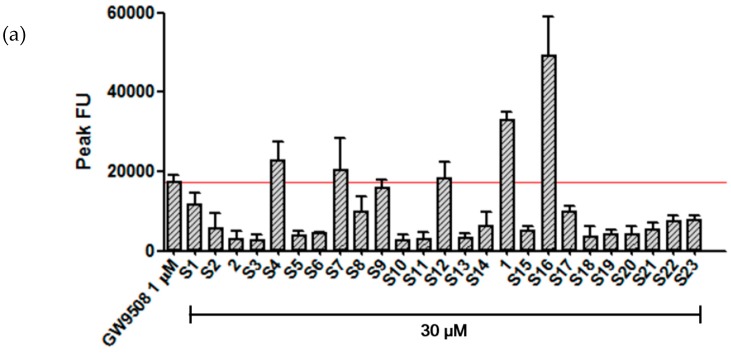

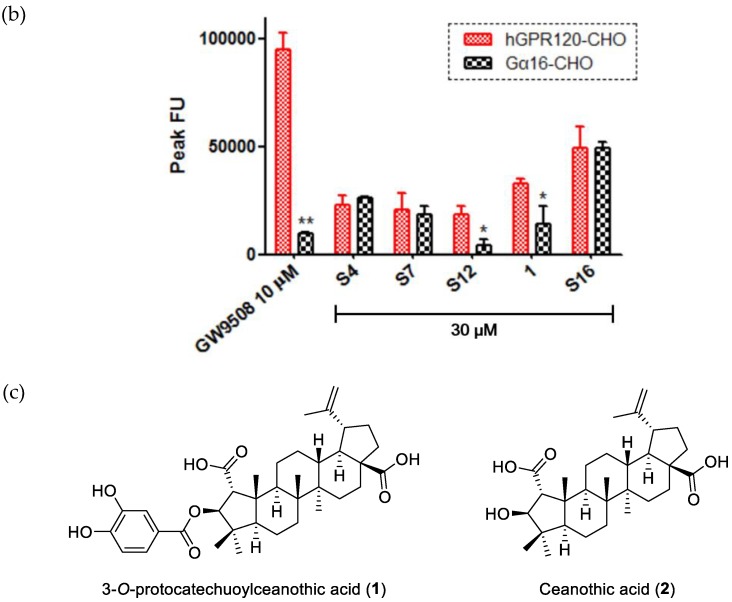
(**a**) GPR120 agonist activity of 25 triterpenoids isolated from *Ziziphus jujuba*. ([Ca^2+^]i in hGPR120-CHO cells); (**b**) Comparison of GPR120 agonist activity between hGPR120-CHO cells and Gα16-CHO cells ([Ca^2+^]i in hGPR120-CHO cells and [Ca^2+^]i in Gα16-CHO cells). The statistical difference between each group was analyzed using a Student’s *t*-test (* *p* < 0.05, ** *p* < 0.001, compared with hGPR120-CHO cells); (**c**) Structures of 3-*O*-protocatechuoylceanothic acid (**1**) and ceanothic acid (**2**).

Omega-3 fatty acids consist of lipophilic carboxylic acid moieties. Since GPR120 is activated by omega-3 fatty acids [2,3,4,5], we assumed that carboxylic acids possessing a relevant lipophilic backbone could also activate GPR120. Consequently, we screened 25 triterpenoids with relevant structural features which were isolated from the traditional medicinal plant *Ziziphus jujuba* (Figure S1) [17]. GPR120 as a GPCR is mainly coupled with Gαq protein [3,4,5]. To investigate the GPR120 agonist activities of the isolated 25 triterpenoids, we examined the Gαq-mediated intracellular calcium ([Ca^2+^]i) release after exposing hGPR120-CHO cells to a known GPR120 agonist, GW-9508, or to the isolated components (Figure 2a) [18]. The intracellular calcium ([Ca^2+^]i) was significantly increased by five natural triterpenoids, namely, **S4**, **S7**, **S12**, 3-*O*-Protocatechuoylceanothic acid (**1**)**,** and **S16**; the other components did not have any significant effects. In order to clarify whether the enhanced [Ca^2+^]i is mediated through GPR120 signaling, we further tested the ligand activity in the Gα16-CHO cells (Figure 2b). Triterpenoids **S4**, **S7**, and **S16** increased [Ca^2+^]i in these cells, and the fluorescence intensity was almost comparable to that observed in the hGPR120-CHO cells. However, triterpenoids **S12** and **1** only marginally increased [Ca^2+^]i in the Gα16-CHO cells, indicating that these components were selective agonists of GPR120. 3-*O*-Protocatechuoylceanothic acid (**1**) was found to be the most potent component based on the [Ca^2+^] FLIPR assay (Figure 2a,b). However, the paucity of 3-*O*-protocatechuoylceanothic acid (**1**) (5.8 mg from 7.5 kg air-dried *Ziziphus jujuba* roots [17]) limited further studies on important aspects such as the medicinal chemistry of this natural and novel GPR120 agonists. Thus, we extensively investigated the synthetic routes for efficiently obtaining 3-*O*-protocatechuoylceanothic acid **1** in sufficient yields. Fortunately, we found a structural similarity between **1** and ceanothic acid (**2**) (Figure 2c), although **2** did not possess any GPR120 agonist activity. Thus, we focused on converting **2** into **1**, as the yield of **2** isolated from *Ziziphus jujuba* (14.5 g from 7.5 kg air-dried *Ziziphus jujuba* roots [17]) was significantly high.

**Figure 3:**
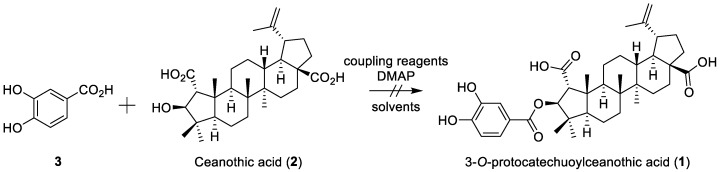
Initial strategy for the direct esterification of ceanothic acid (**2**) and benzoic acid **3**.

We anticipated that the direct esterification of **2** (Figure 3) would conveniently provide **1**. In addition, we anticipated some structural variation of the benzene-moiety upon esterification, as it was identified as a key unit for the GPR120 agonist activity of **1**. However, the semi-synthesis of **1** via direct esterification of **2** with benzoic acid **3** was not successful. Numerous attempts to achieve the esterification of **2** under various reaction conditions consistently resulted in complex mixtures, including self-polymerized products and regioisomers.

**Scheme 1:**
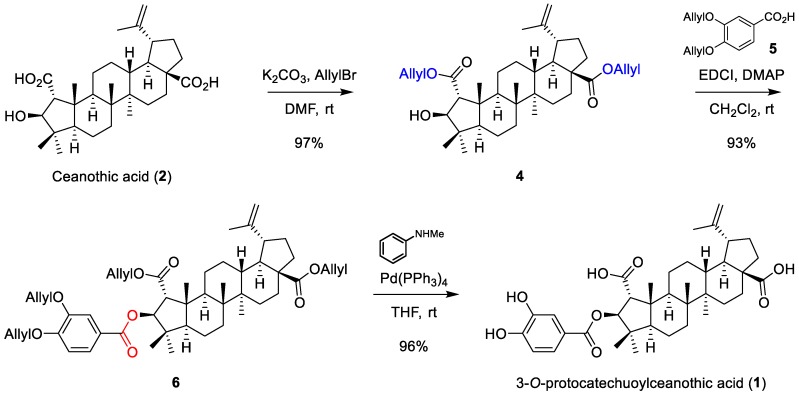
Semi-synthesis of 3-*O*-protocatechuoylceanothic acid (**1**) from ceanothic acid (**2**).

Following the failure of direct esterification, we focused on the facile and concomitant protection of both carboxylic acid of **2** and the catechol moiety of **3**, before being subjected to esterification. For this purpose, we first protected the two carboxylic acid groups on **2** without etherification of the free hydroxyl group. In addition, the benzoyl ester group between **2** and **3** should be tolerated during the late deprotection of these two esters. Thus, we proceeded with the allyl protection of the two carboxylic acids because it could preserve the chemoselectivity during both protection and deprotection of the acids. In addition, the allyl-protected acid and catechol can be globally deprotected at the final stage. The synthesis of **1** commenced with allyl protection of the two carboxylic acids of **2**, as shown in Scheme 1. The allyl ester **4** was efficiently formed, with the free hydroxyl group remaining intact. 1-Ethyl-3-(3-dimethylaminopropyl)carbodiimide (EDCI)-mediated coupling of alcohol **4** and benzoic acid **5** [19] produced *tetra*-allyl protected ester **6** in 90% yield in two steps. Finally, global allyl-deprotection of ester **6** with Pd(0) catalyst in the presence of *N*-Me-aniline effectively produced **1** in 96% yield. Unfortunately, the NMR spectrum of **1** synthesized by us was not identical to its reported spectral data [17]. However, to our delight, the NMR spectrum of alternatively isolated **1** [17] from *Ziziphus jujuba* roots was identical to that of chemically synthesized **1**. Moreover, the HPLC analysis also supported that both synthesized and isolated **1** were identical. Thus, herein, we also report the corrected NMR chemical shifts of **1** (Table S1).

## 3. Materials and Methods 

### 3.1. Biology

#### Analysis of GPR120 Ligand Activity

The increase in GPR120-mediated intracellular Ca^2+^ was measured by the [Ca^2+^] FLIPR assay as described previously [18]. Briefly, CHO cells overexpressing hGPR120 (hGPR120-CHO) and Gα16 (Gα16-CHO) were seeded (5 × 10^4^ cells of each type) in 96-well plates and cultured overnight. Then, the final volume was adjusted to 200 μL using the assay buffer of the [Ca^2+^] FLIPR assay kit (Molecular Devices). hGPR120-CHO cells were incubated with 1 or 10 µM GW-9508 (positive control) or 28 natural triterpenoids (30 µM) for 1 h at 37 °C. Using FlexStation, the intracellular Ca^2+^ concentration was measured for 3 min at intervals of 2.5 s, after setting the excitation, emission, and cut-off at 485, 525, and 515 nm, respectively. Data are expressed as the mean ± standard deviation. The statistical difference between each group was analyzed using a Student’s *t*-test. 

### 3.2. Chemistry

#### 3.2.1. General Information

Unless noted otherwise, all starting materials and reagents were obtained from commercial suppliers and were used without further purification. Tetrahydrofuran was distilled from sodium benzophenone ketyl. Dichloromethane was freshly distilled from calcium hydride. All solvents used for routine isolation of products and chromatography were reagent grade and glass distilled. Reaction flasks were dried at 100 °C. Air and moisture sensitive reactions were performed under an argon atmosphere. Flash column chromatography was performed using silica gel 60 (230–400 mesh, Merck, Kenilworth, NJ, USA) with the indicated solvents. Thin-layer chromatography was performed using 0.25 mm silica gel plates (Merck, Kenilworth, NJ, USA). Optical rotations were measured with JASCO P-2000 digital polarimeter (Tokyo, Japan) at ambient temperature using cylindrical cell of 10 mm or 100 mm pathlength. Infrared spectra were recorded on a JASCO FT-IR-4200 spectrometer (Tokyo, Japan). High resolution mass spectra were obtained with Agilent Q TOF 6530 (Santa Clara, CA, USA). ^1^H and ^13^C NMR spectra were recorded using BRUKER AVANCE-800 (Billerica, MA, USA). Chemical shifts are expressed in parts per million (ppm, *δ*) downfield from tetramethylsilane and are referenced to the deuterated solvent (CHCl_3_, ^1^H *δ* 7.24, ^13^C *δ* 77.0; pyridine, ^1^H *δ* 8.69, 7.55 and 7.18, ^13^C *δ* 149.7, 135.5 and 123.4). ^1^H-*N*MR data were reported in the order of chemical shift, multiplicity (s, singlet; d, doublet; t, triplet; q, quartet; m, multiplet and/or multiple resonances), coupling constant in hertz (Hz) and number of protons. High performance liquid chromatography (HPLC) experiments were performed with Waters 1525 binary pump (Milford, MA, USA) and Waters 2489 UV/Visible detector (Milford, MA, USA) at 35 °C.

#### 3.2.2. Experimental Part

*Diallyl (1R,2S,5aR,5bR,7aS,10R,12bR)-2-hydroxy-3,3,5a,5b,12b-pentamethyl-10-(prop-1-en-2- yl)octadecahydrodicyclopenta[a,i]phenanthrene-1,7a(1H)-dicarboxylate* (**4**). To a solution of ceanothic aid (**2**) (0.45 g, 0.92 mmol) in dry DMF (9.2 mL) were added allyl bromide (0.40 mL, 4.62 mmol) and K_2_CO_3_ (0.64 g, 4.62 mmol) at room temperature. After stirring overnight, the reaction mixture was quenched with 1N HCl and extracted with Et_2_O (10 mL × 3). The combined organic layer was washed with brine, dried over MgSO_4_ and concentrated in vacuo. The residue was purified by flash column chromatography (EtOAc/Hexane = 1:10) to give 0.51 g (97%) of ester **4** as white amorphous powder. [α]_D_^20^ = +25.31 (*c* 1.00, CHCl_3_); ^1^H NMR (800 MHz, CDCl_3_) *δ* 5.90 (ddt, *J* = 17.0, 10.4, 6.2 Hz, 1H), 5.89 (ddt, *J* = 17.2, 10.5, 5.7 Hz, 1H), 5.31 (ddt, *J* = 17.2, 1.6, 1.5 Hz, 1H), 5.30 (ddt, *J* = 17.2, 1.6, 1.5 Hz, 1H), 5.20 (dd, *J* = 10.3, 1.0 Hz, 1H), 5.20 (dd, *J* = 10.4, 1.2 Hz, 1H), 4.69 (d, *J* = 1.8 Hz, 1H), 4.58–4.51 (m, 4H), 4.46 (ddt, *J* = 12.8, 6.1, 1.1 Hz, 1H), 4.14 (s, 1H), 2.96 (td, *J* = 10.8, 5.1 Hz, 1H), 2.58 (s, 1H), 2.25–2.22 (m, 1H), 2.15 (td, *J* = 12.7, 3.7 Hz, 1H), 1.92–1.82 (m, 2H), 1.69 (s, 1H), 1.64 (s, 3H), 1.61 (dd, *J* = 11.7, 3.1 Hz, 1H), 1.61–1.58 (m, 1H), 1.53 (t, *J* = 11.4 Hz, 1H), 1.48–1.45 (m, 1H), 1.43 (s, 1H), 1.42–1.29 (m, 9H), 1.12–1.09 (m, 1H), 1.09 (s, 3H), 0.94–0.91 (m, 1H), 1.04 (s, 3H), 0.90 (s, 3H), 0.88 (s, 6H); ^13^C NMR (200 MHz, CDCl_3_) *δ* 175.7, 174.5, 150.3, 132.5, 132.1, 119.1, 118.1, 109.6, 84.9, 65.5, 65.4, 64.6, 56.6, 56.5, 49.6, 49.5, 46.9, 44.6, 43.3, 42.9, 41.7, 38.5, 37.0, 33.9, 32.2, 30.7, 30.5, 29.7, 25.2, 23.4, 19.4, 19.2, 18.5, 18.4, 16.5, 14.7; IR (thin film, neat) *ν*_max_ 2947, 2869, 1722, 1644, 1455, 1173, 987, 932 cm^−1^; LR-MS (ESI+) *m/z* 584 (M + NH_4_^+^); HR-MS (ESI+) calcd for C_36_H_58_NO_5_ (M + NH_4_^+^) 584.4310; found 584.4312.

*Diallyl (1R,2S,5aR,5bR,7aS,10R,12bR)-2-((3,4-bis(allyloxy)benzoyl)oxy)-3,3,5a,5b,12b-pentamethyl-10- (prop-1-en-2-yl)octadecahydrodicyclopenta[a,i]phenanthrene-1,7a(1H)-dicarboxylate* (**6**). To a solution of diester alcohol **4** (0.45 g, 0.79 mmol), benzoic acid **5** (0.24 g, 1.03 mmol), and DMAP (0.19 g, 1.59 mmol) in CH_2_Cl_2_ (7.9 mL) was added EDC∙HCl (0.30 g, 1.59 mmol) at room temperature. After stirring for 2 h, the reaction mixture was quenched with 1N HCl and extracted with CH_2_Cl_2_ (10 mL × 3). The combined organic layer was washed with aqueous NaHCO_3_, dried over MgSO_4_, and concentrated in vacuo. The residue was purified by flash column chromatography (EtOAc/Hexane = 1:20) to give 0.58 g (93%) of ester **6** as a colorless oil. [α]_D_^20^ = −22.23 (*c* 1.00, CHCl_3_); ^1^H NMR (800 MHz, CDCl_3_) *δ* 7.58 (dd, *J* = 8.4, 2.0 Hz, 1H), 7.52 (d, *J* = 2.0 Hz, 1H), 6.86 (d, *J* = 8.5 Hz, 1H), 6.04 (ddt, *J* = 17.1, 10.6, 5.3 Hz, 2H), 5.93 (ddt, *J* = 17.1, 10.5, 6.2 Hz, 1H), 5.90 (ddt, *J* = 17.2, 10.5, 5.7 Hz, 1H), 5.41 (dtd, *J* = 17.3, 1.5, 1.4 Hz, 1H), 5.40 (dq, *J* = 17.3, 1.5 Hz, 1H), 5.33 (dd, *J* = 9.7, 1.4 Hz, 1H), 5.31 (ddt, *J* = 10.1, 2.4, 1.3 Hz, 1H), 5.29 (dd, *J* = 10.6, 1.4 Hz, 1H), 5.27 (s, 1H), 5.27 (dd, *J* = 10.6, 1.4 Hz, 1H), 5.22 (dd, *J* = 10.3, 1.1 Hz, 1H), 5.21 (dd, *J* = 10.4, 1.3 Hz, 1H), 4.70 (d, *J* = 1.6 Hz, 1H), 4.64 (dt, *J* = 5.2, 1.4 Hz, 2H), 4.62 (dt, *J* = 5.3, 1.5 Hz, 2H), 4.62–4.60 (m, 1H), 4.59–4.56 (m, 2H), 4.55–4.49 (m, 2H), 2.96 (td, *J* = 11.0, 5.0 Hz, 1H), 2.72 (s, 1H), 2.25 (dd, *J* = 12.2, 3.3 Hz, 1H), 2.18 (td, *J* = 12.7, 3.6 Hz, 1H), 1.92–1.85 (m, 2H), 1.72 (dd, *J* = 10.2, 4.9 Hz, 1H), 1.65 (s, 3H), 1.61 (dd, *J* = 13.3, 2.3 Hz, 1H), 1.53 (d, *J* = 11.4 Hz, 1H), 1.52–1.48 (m, 1H), 1.48–1.36, (m, 7H), 1.36–1.31 (m, 3H), 1.24 (s, 3H), 1.14–1.10 (m, 1H), 1.09 (s, 3H), 0.99–0.95 (m, 1H), 0.94 (s, 3H), 0.91 (s, 3H), 0.90 (s, 3H); ^13^C NMR (200 MHz, CDCl_3_) *δ* 175.7, 173.5, 165.6, 152.5, 150.3, 147.9, 133.0, 132.6, 132.5, 132.0, 123.5, 122.8, 119.3, 118.1 (2C), 118.0, 114.6, 112.4, 109.6, 85.6, 69.8, 69.6, 65.7, 64.6, 63.2, 56.5, 56.4, 49.7, 49. 4, 46.9, 44.7, 43.3, 42.9, 41.7, 38.5, 37. 0, 33.9, 32.2, 30.6, 30.2, 29.8, 25.2, 23.3, 19.9, 19.4, 18.4, 18.2, 16.5, 14.7; IR (thin film, neat) *ν*_max_ 2946, 2869, 1723, 1600, 1427, 1270, 1203, 1131, 986, 929 cm^−1^; LR-MS (ESI+) *m/z* 783 (M + H^+^); HR-MS (ESI+) calcd for C_49_H_67_O_8_ (M + H^+^) 783.4830; found 783.4827. 

*3-*O*-Protocatechuoylceanothic Acid* (**1**). To a solution of ester **6** (0.50 g, 0.64 mmol) and *N*-methylaniline (0.35 mL, 3.19 mmol) in dry THF (6.4 mL) was added Pd(PPh_3_)_4_ (0.15 g, 0.13 mmol) at room temperature. After stirring for 2 h, the reaction mixture was quenched with 1*N* HCl and extracted with EtOAc (10 mL × 3). The combined organic layer was washed with brine, dried over MgSO_4_, and concentrated in vacuo. The residue was purified by flash column chromatography (EtOAc/Hexane = 1:3 to CH_2_Cl_2_/MeOH = 16:1) to give 0.38 g (96%) of 3-*O*-Protocatechuoylceanothic Acid (**1**) as yellowish powder. [α]_D_^20^ = −17.19 (*c* 1.10, CHCl_3_); ^1^H NMR (800 MHz, Pyridine-*d*_5_) *δ* 8.07 (d, *J* = 2.1 Hz, 1H), 7.86 (dd, *J* = 8.2, 2.1 Hz, 1H), 7.28 (d, *J* = 8.2 Hz, 1H), 5.89 (s, 1H), 4.82 (s, 1H), 4.62 (s, 1H), 3.46 (td, *J* = 10.8, 5.0 Hz, 1H), 3.07 (s, 1H), 2.74 (td, *J* = 12.7, 3.6 Hz, 1H), 2.56 (dt, *J* = 12.8, 3.3 Hz, 1H), 2.23–2.17 (m, 2H), 2.13–2.08 (m, 2H), 2.07 (dd, *J* = 12.5, 2.6 Hz, 1H), 1.97–1.93 (m, 1H), 1.85 (td, *J* = 13.5, 3.6 Hz, 1H), 1.65 (t, *J* = 11.3 Hz, 1H), 1.63 (s, 3H), 1.54 (qd, *J* = 12.8, 4.3 Hz, 1H), 1.52–1.49 (m, 1H), 1.49 (s, 3H), 1.47–1.42 (m, 3H), 1.39–1.35 (m, 3H), 1.31 (qd, *J* = 13.1, 4.3 Hz, 1H), 1.18 (dd, *J* = 13.7, 3.0 Hz, 1H), 1.12 (s, 3H), 1.09 (s, 3H), 1.02 (s, 3H), 1.00 (s, 3H); ^13^C NMR (200 MHz, Pyridine-*d*_5_) *δ* 178.8, 176.6, 166.3, 152.5, 151.0, 147.0, 123.0, 122.1, 117.5, 116.2, 109.7, 85.6, 64.0, 56.7, 56.5, 49.5, 49.4, 47.4, 45.2, 43.6, 43.4, 42.0, 38.9, 37.4, 34.5, 32.8, 31.2, 30.5, 30.4, 26.1, 24.1, 20.1, 19.5, 18.6, 18.4, 16.9, 14.9; IR (thin film, neat) *ν*_max_ 3686, 2946, 2871, 1697, 1523, 1066, 1038, 1018 cm^−1^; LR-MS (ESI−) *m/z* 621 (M − H^+^); HR-MS (ESI−) calcd for C_37_H_49_O_8_ (M − H^+^) 621.3433; found 621.3443.

## 4. Conclusions

In summary, we identified a novel and natural GPR120 agonist, 3-*O*-protocatechuoylceanothic acid (**1**), which is a ceanothane-type triterpene. We also synthesized **1** from ceanothic acid (**2**) in three steps, which enabled us to overcome the potential difficulties due to the paucity of **1**. The key features of our synthesis include the elaborate acid protection of di-acid **2** and catechol and the facile global deprotection of the four allyl groups of **6**, keeping the other functional groups intact. Further evaluation of the biological activity and medicinal chemistry of 3-*O*-protocatechuoylceanothic acid (**1**), including the structure-activity relationship, is underway.

## Figures and Tables

**Table:** 

Coupling reagents	Solvents	Results
DCC, EDCI, HATU, DEPBT, PyBOP	CH_2_Cl_2_	No reaction (>90% of **2**)
	DMF	40–80% of **2** and complex mixture
	CH_2_Cl_2_:DMF = 2:1	Complex mixture (none of **2**)

## Supplementary Materials

The following are available online. Figure S1. Structures of the triterpenoids; Table S1. Corrected NMR chemical shifts of 3-*O*-protocatechuoylceanothic acid (1); Table S2. [Ca^2+^]i in hGPR120-CHO cells; Table S3. Comparison of [Ca^2+^]i between hGPR120-CHO cells and Gα16-CHO cells; The ^1^H and ^13^C NMR spectra of **4**, **6** and **1** and HPLC analysis of synthetic and natural **1**.
