# Supplementary material for: Identification and Semi-Synthesis of 3-O-Protocatechuoylceanothic Acid, a Novel and Natural GPR120 Agonist
Source: Molecules. 2019 Sep 26;24(19):3487. doi: 10.3390/molecules24193487 (PMC6804280; doi:10.3390/molecules24193487)

Supporting Information

Identification and Semi-Synthesis of 3-*O*-Protocatechuoylceanothic Acid, a Novel and Natural GPR120 Agonist[†]

Changjin Lim [1], Jung Gyu Park [2], Kyo Bin Kang [3], and Young-Ger Suh [1,2,*]

[1] *College of Pharmacy, CHA University, 120 Haeryong-ro, Pocheon 11160, Gyeonggi-do, Korea*
[2] *College of Pharmacy and Research Institute of Pharmaceutical Sciences, Seoul National University, 1 Gwanak-ro, Gwanak-gu, Seoul 08826, Korea*
[3] *Research Institute of Pharmaceutical Sciences, College of Pharmacy, Sookmyung Women's University, Seoul 04310, Korea*
[†] *In memory of late professor Sang Hyun Sung*

Correspondence: ygsuh@cha.ac.kr (or ygsuh@snu.ac.kr) Tel.: +82-31-850-9300

Table of Contents

I. Structures of the Triterpenoids ······ S2

II. NMR chemical shifts of 3-*O*-protocatechuoylceanothic acid (**1**) ······ S3

III. [Ca^{2+}] FLIPR Assay Data ······ S4

IV. ^{1}H- and ^{13}C-NMR Spectra ······ S5

V. HPLC Analysis for 3-*O*-Protocatechuoylceanothic Acid (**1**) ······ S9

I. Structures of the Triterpenoids

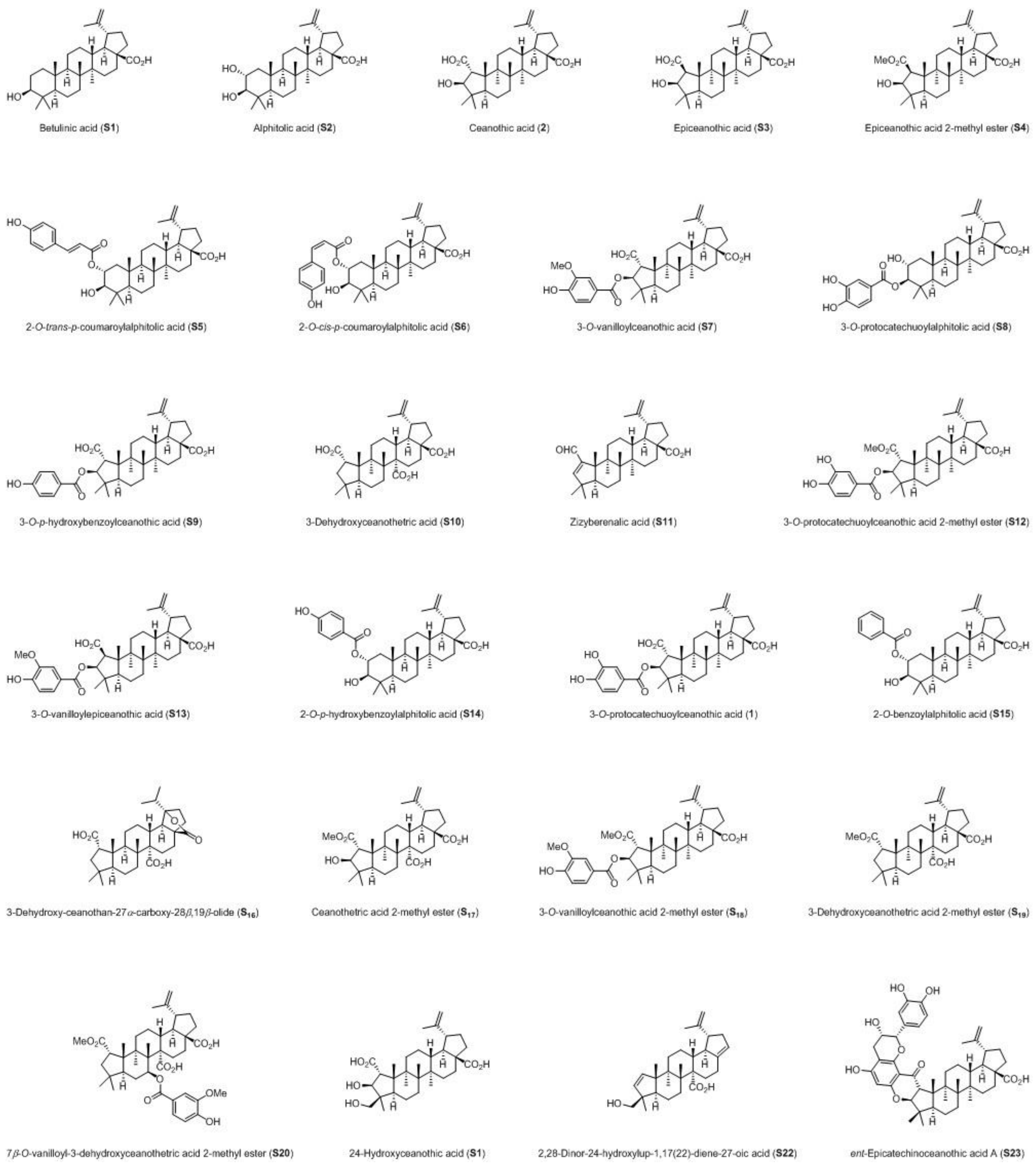

Figure S1. Structures of the triterpenoids

II. NMR chemical shifts of 3-*O*-protocatechuoylceanothic acid (1)

Table 1. Corrected NMR chemical shifts of 3-*O*-protocatechuoylceanothic acid (**1**)[a]

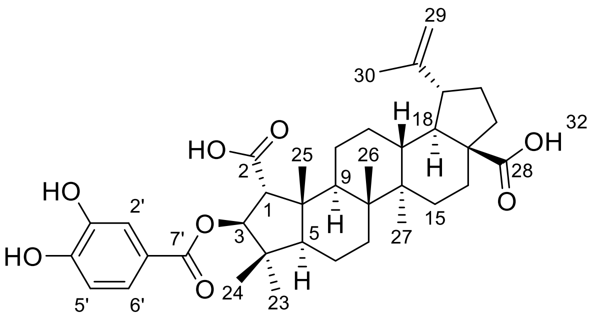

3-*O*-protocatechuoylceanothic acid (**1**)

No.	^{1}H-NMR[b]	No.	^{1}H-NMR[b]	No.	^{13}C-NMR[c]	No.[b]	^{13}C-NMR[c]
1	3.07, s	19	3.46, td (10.8, 5.0)	1	64.0	20	151.0
3	5.89, s	21a	2.23–2.17, m	2	176.6	21	31.2
5	2.13–2.08, m	21b	1.47–1.42, m	3	85.6	22	37.4
6	1.39–1.35, m	22a	2.23–2.17, m	4	43.6	23	30.5
7a	1.47–1.42, m	22b	1.52–1.49, m	5	56.7	24	20.1
7b	1.39–1.35, m	23	1.49, s	6	18.6	25	18.4
9	2.07, dd (12.5, 2.6)	24	1.03, s	7	34.5	26	16.9
11a	2.13–2.08, m	25	1.12, s	8	43.4	27	14.9
11b	1.54, qd (12.8, 4.3)	26	1.09, s	9	45.2	28	178.8
12a	1.97–1.93, m	27	1.00, s	10	49.4	29	109.7
12b	1.31, qd (13.1, 4.3)	29a	4.82, s	11	24.1	30	19.5
13	2.74, td (12.7, 3.6)	29b	4.62, s	12	26.1	1′	122.1
15a	1.85, td (13.5, 3.6)	30	1.63, s	13	38.9	2′	117.5
15b	1.18, dd (13.7, 3.0)	2′	8.07, d (2.1)	14	42.0	3′	152.5
16a	2.56, dt (12.8, 3.3)	5′	7.28, d (8.2)	15	30.4	4′	147.0
16b	1.47–1.42, m	6′	7.86, dd (8.2, 2.1)	16	32.8	5′	116.2
18	1.65, t (11.3)			17	56.5	6′	123.0
				18	49.5	7′	166.3
				19	47.4		

[a]Recorded at 800 MHz. Spectra were measured in pyridine-d_5;
[c]chemical shift in ppm and multiplicity (coupling constant in hertz (Hz));
[d]chemical shift in ppm

III. [Ca^{2+}] FLIPR Assay Data

Table S2. [Ca^{2+}]i in hGPR120-CHO cells

Concentration	Compound	Peak FU #1	Peak FU #2	Peak FU #3	Peak FU #4	Average	S.D
1μM	GW9508	17076.05	18994.03	14877.98	18531.59	17369.91	1851.426
	S1	14767.48	8194.688	10654.04	13178.38	11698.65	2885.381
	S2	5972.58	9853.587	404.569	6403.341	5658.519	3909.645
	2	2898.567	777.84	5556.189	3260.223	3123.205	1956.986
	S3	2063.313	3819.886	1187.656	3969.781	2760.159	1359.481
	S4	20335.81	19582.18	22221.97	29550.14	22922.53	4555.768
	S5	2853.537	3103.732	4949.489	4912.807	3954.891	1132.001
	S6	4532.879	4430.379	4793.11	4338.657	4523.756	196.3124
	S7	31757.23	20682.43	15753.34	13876.57	20517.39	8024.087
	S8	9299.125	13746.46	5001.649	11389.01	9859.061	3713.067
	S9	18246.87	14383.85	14092.64	17016.85	15935.05	2026.121
	S10	1600.75	3593.974	4368.375	1444.539	2751.91	1455.612
	S11	2608.098	4909.324	1323.676	3660.645	3125.436	1525.635
30μM	S12	15520	20476.34	14157.02	22864.59	18254.49	4101.07
	S13	2151.511	3963.527	3033.328	4453.602	3400.492	1019.937
	S14	4834.398	11662.74	4220.512	4748.363	6366.503	3541.239
	1	35766.83	31913.7	31440.34	32777.97	32974.71	1942.05
	S15	6114.27	5570.329	5601.178	3865.609	5287.847	980.4265
	S16	41684.05	42687.96	50272.39	62611.62	49314.01	9658.602
	S17	9300.937	10720.66	8217.664	11139.01	9844.568	1339.834
	S18	4063.215	0	5887.253	4818.085	3692.138	2572.668
	S19	4463.89	4916.318	4923.184	2932.855	4309.062	942.3062
	S20	2381.556	3690.635	6987.603	4513.111	4393.226	1939.554
	S21	2957.117	7278.951	6133.255	5089.824	5364.787	1837.303
	S22	6358.697	7374.226	6973.231	9664.489	7592.661	1442.977
	S23	8263.668	8264.023	6236.505	8395.536	7789.933	1037.478

Table S3. Comparison of [Ca^{2+}]i between hGPR120-CHO cells and Gα16-CHO cells

Concentration	Compound	hGPR120-CHO			
10μM	GW9508	86443.91	91064.44	102253	100209
	S4	20335.81	19582.18	22221.97	29550.14
	S7	31757.23	20682.43	15753.34	13876.57
30μM	S12	15520	20476.34	14157.02	22864.59
	1	35766.83	31913.7	31440.34	32777.97
	S16	41684.05	42687.96	50272.39	62611.62
**Concentration**	**Compound**	**Gα16-CHO**			
10μM	GW9508	10346.92	9348.441	10164.7	9881.233
	S4	25987.67	27002.4	25991.07	25006.68
	S7	13854.09	16421.07	19914.55	22860.2
30μM	S12	3935.503	6846.605	5234.308	0
	1	2629.238	15312.43	18946.57	20159.61
	S16	46862.36	50376.48	52758.3	46911.38

IV. ^{1}H- and ^{13}C-NMR Spectra

^{1}H-NMR Spectra of **4** (800 MHz, $CDCl_3$)

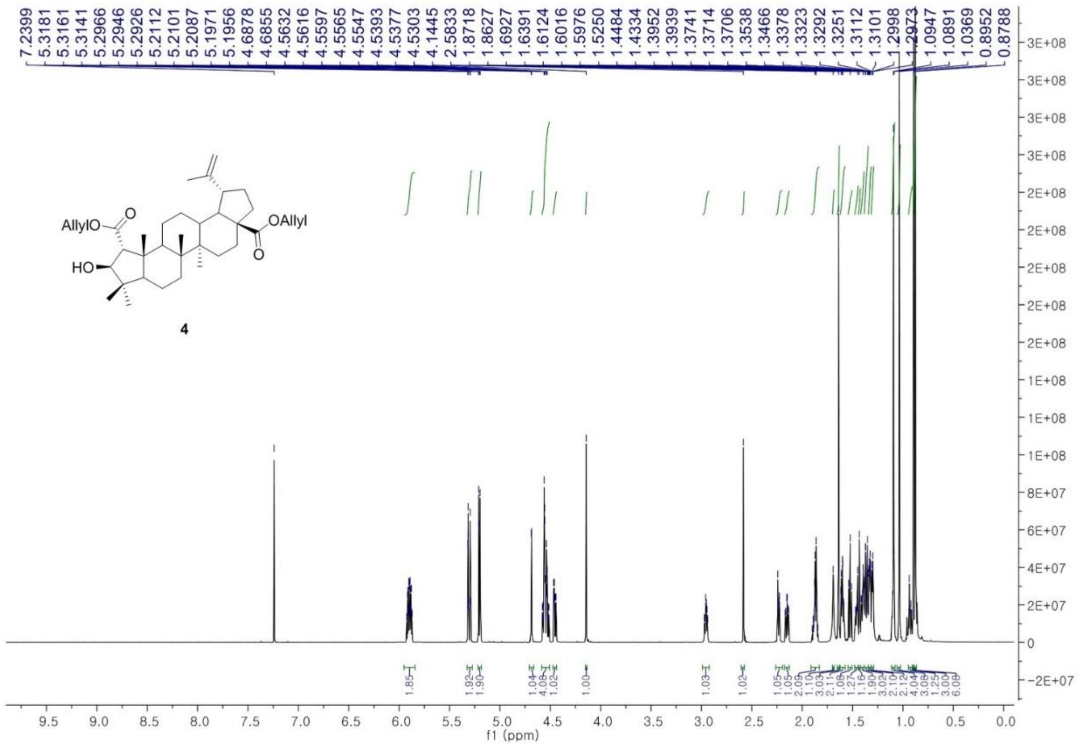

^{13}C-NMR Spectra of **4** (200 MHz, $CDCl_3$)

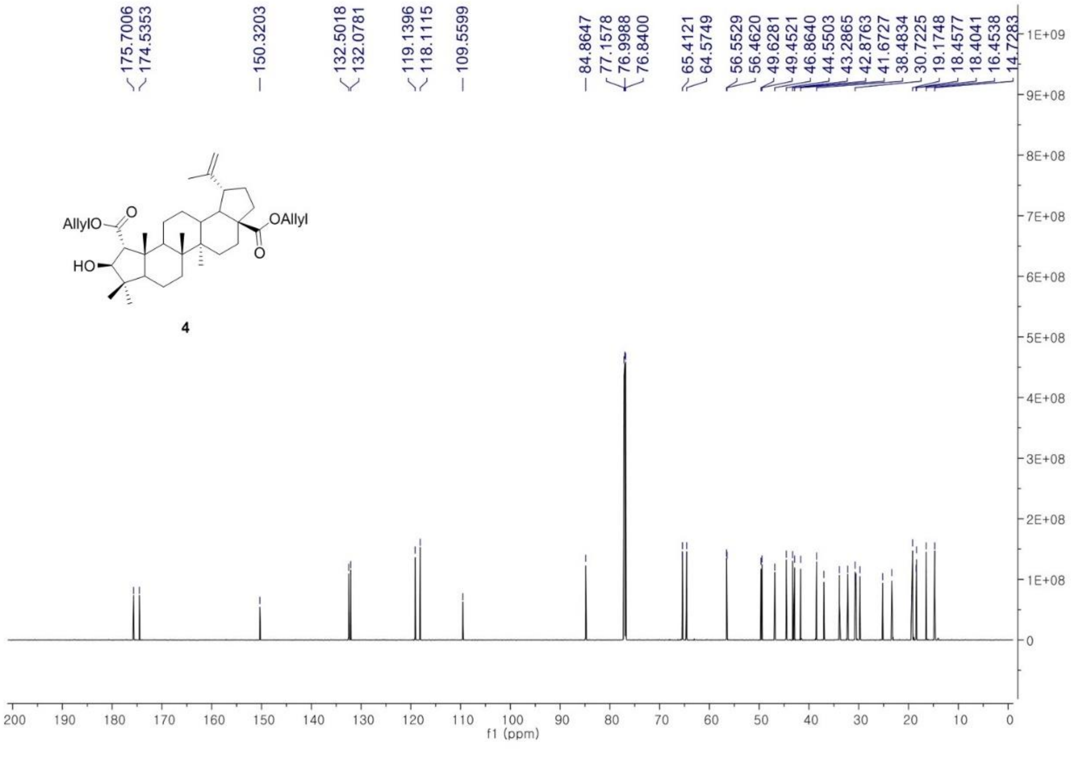

^{1}H-NMR Spectra of **6** (800 MHz, $CDCl_3$)

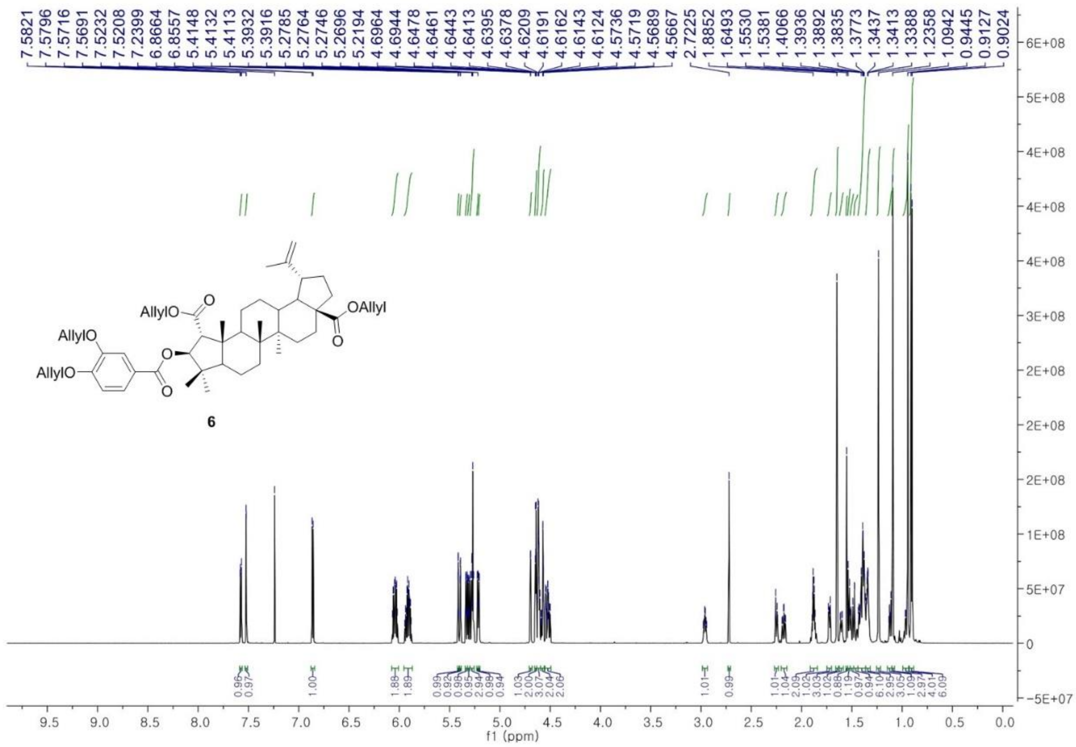

^{13}C-NMR Spectra of **1** (200 MHz, $CDCl_3$)

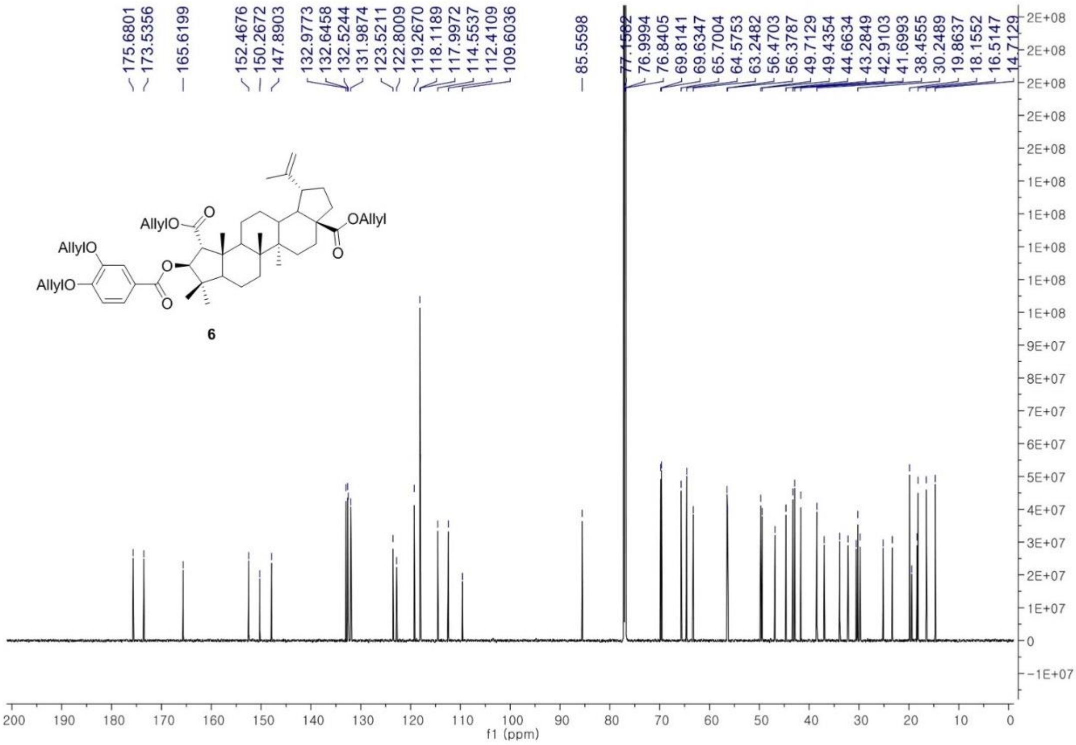

^{1}H-NMR Spectra of Synthetic 3-*O*-Protocatechuoylceanothic Acid (**1**) (800 MHz, Pyridine-d_5)

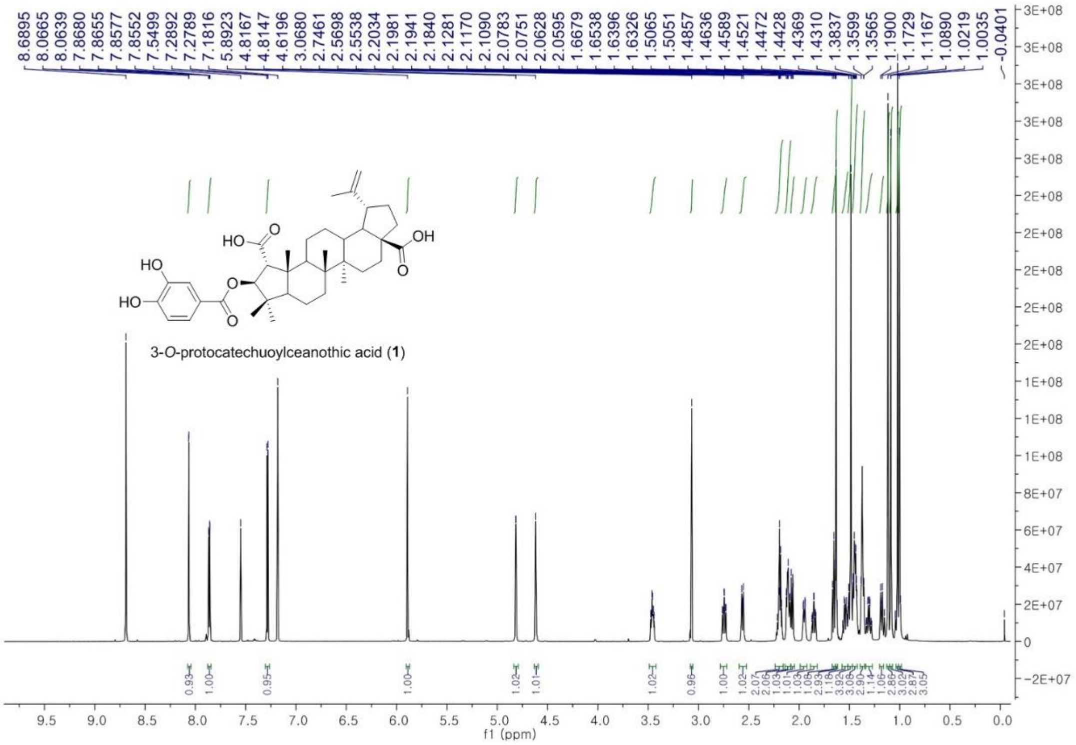

^{1}H-NMR Spectra of Natural 3-*O*-Protocatechuoylceanothic Acid (**1**) (800 MHz, Pyridine-d_5)

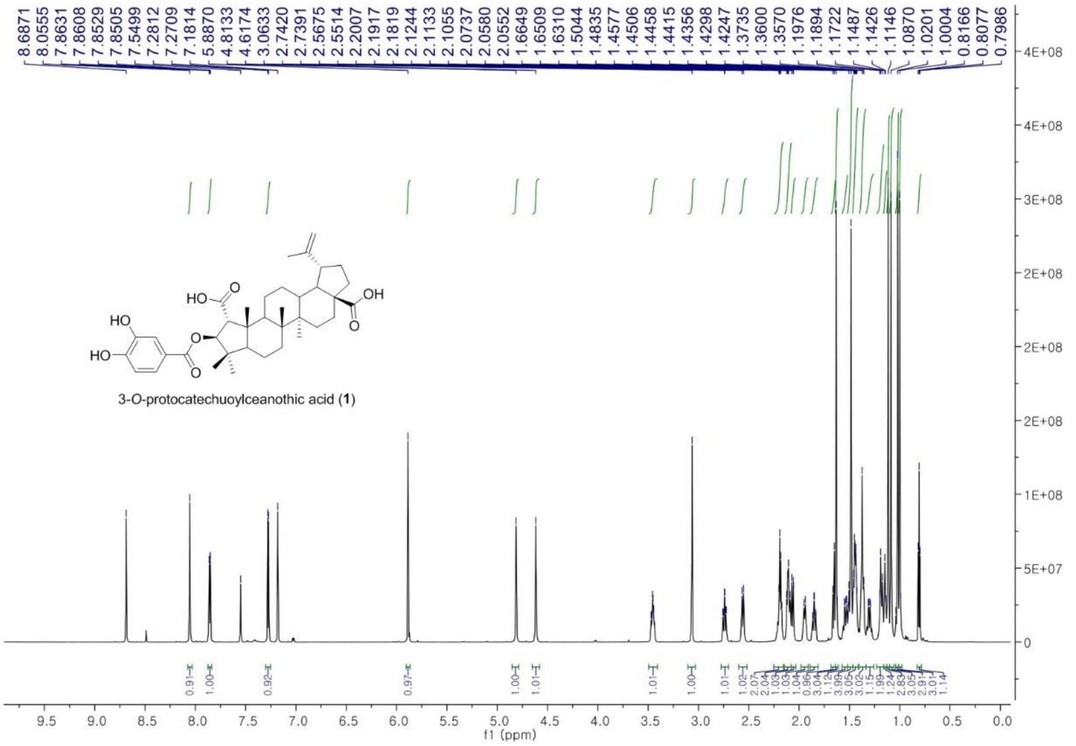

^{13}C-NMR Spectra of Synthetic 3-*O*-Protocatechuoylceanothic Acid (**1**) (200 MHz, Pyridine-d_5)

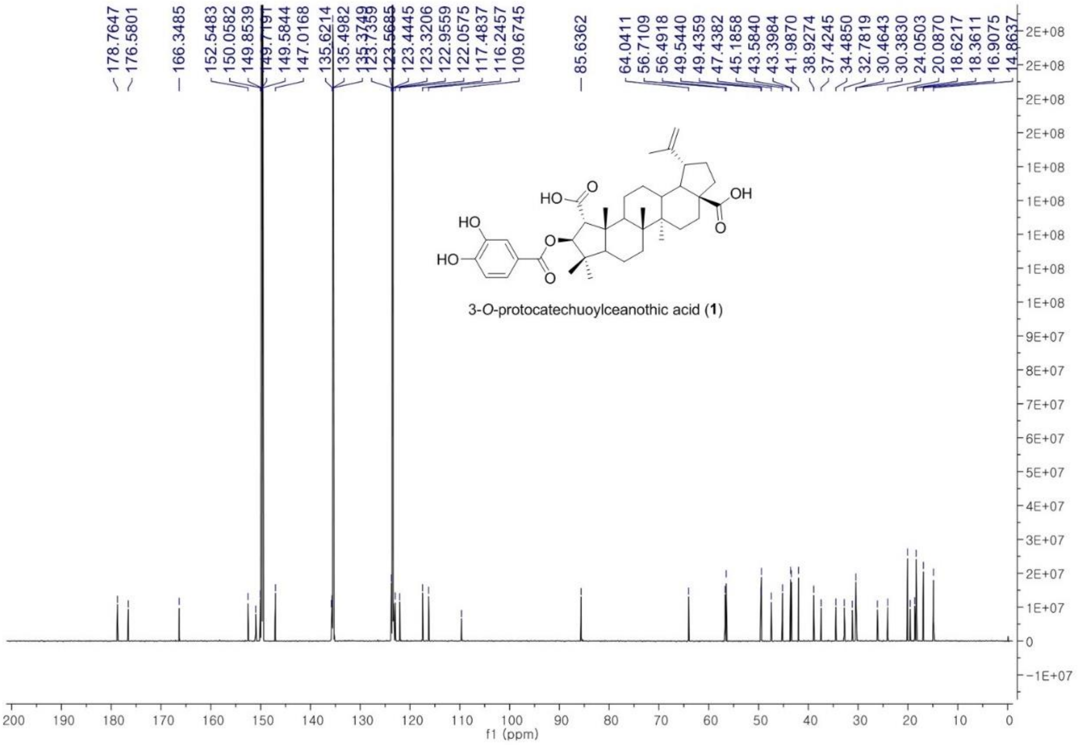

^{13}C-NMR Spectra of Natural 3-*O*-Protocatechuoylceanothic Acid (**1**) (200 MHz, Pyridine-d_5)

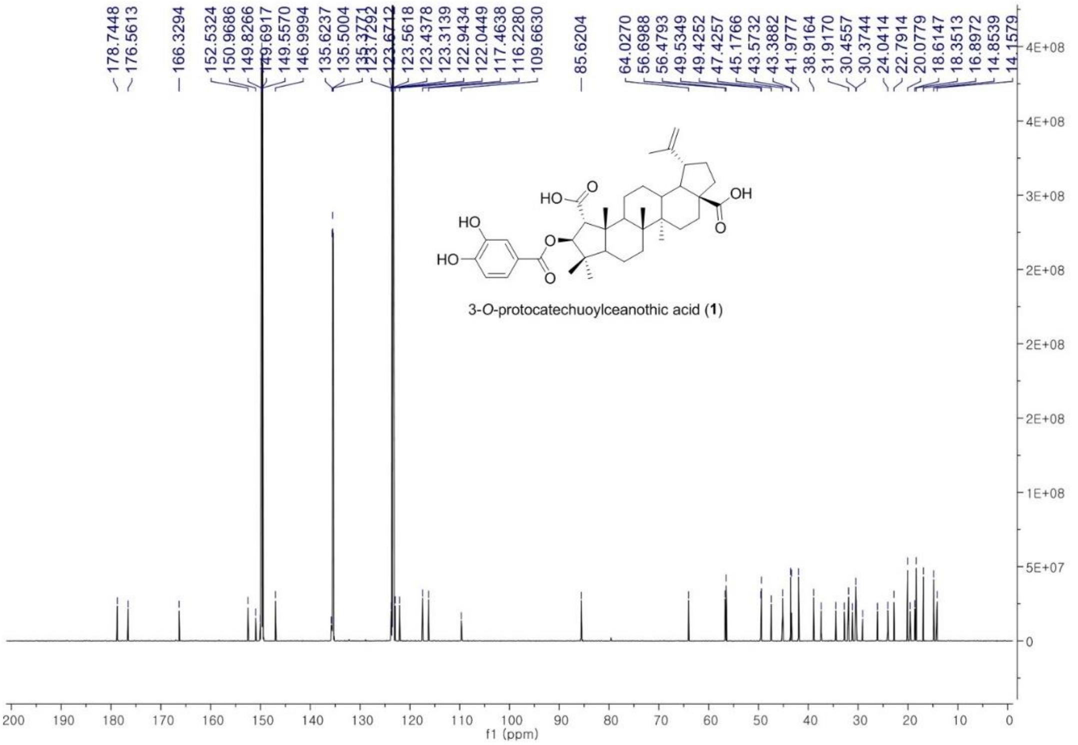

V. HPLC Analysis for 3-*O*-Protocatechuoylceanothic Acid (1)

All chromatograms were obtained via following conditions : Column, Pursuits XRs 5 C18 250 × 10.0 mm; detection, UV 210 nm; Mobile phase, MeCN/H_2O = 70% : 30% (flow rate, 2.0 mL/min)

Natural 3-*O*-Protocatechuoylceanothic Acid (**1**)

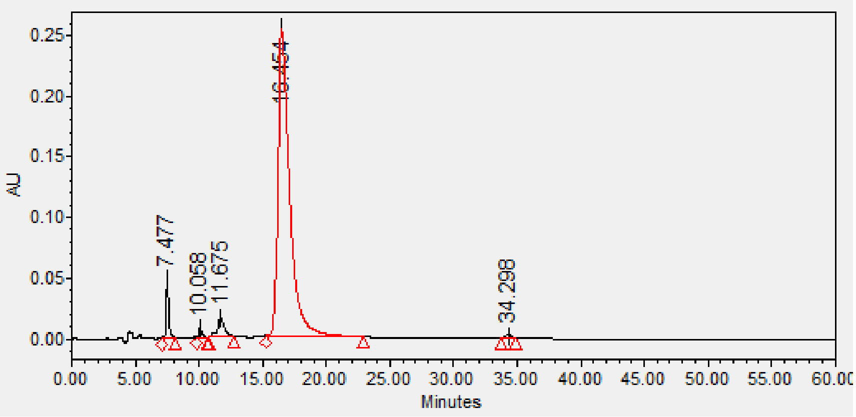

Synthetic 3-*O*-Protocatechuoylceanothic Acid (**1**)

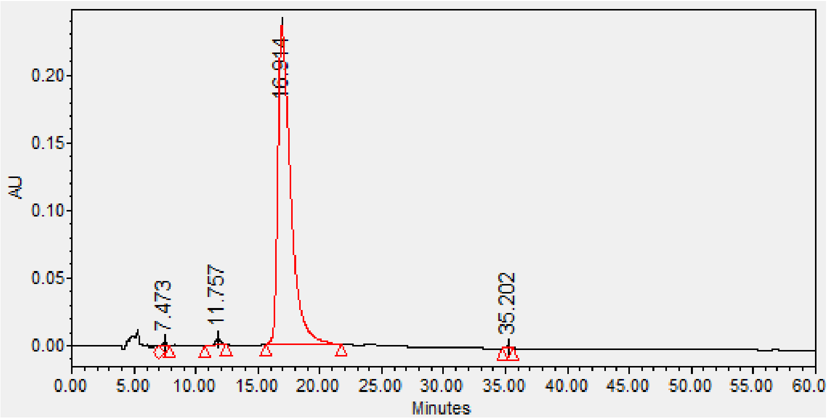

Co-injection of natural and synthetic 3-*O*-Protocatechuoylceanothic Acid (**1**)

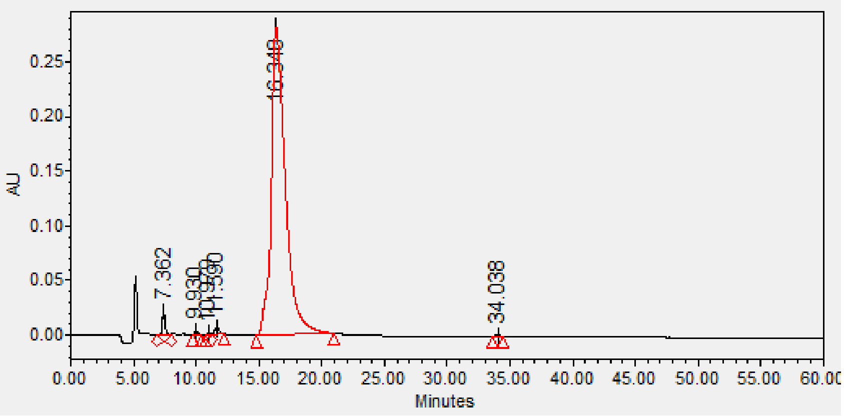

Supplement: Supplementary file 1 [file molecules-24-03487-s001.pdf]
